# Broadband THz absorption spectrometer based on excitonic nonlinear optical effects

**DOI:** 10.1038/s41377-019-0137-y

**Published:** 2019-03-13

**Authors:** Avan Majeed, Pavlo Ivanov, Benjamin Stevens, Edmund Clarke, Iain Butler, David Childs, Osamu Kojima, Richard Hogg

**Affiliations:** 1 Department of Electronic and Electrical Engineering, University of Sheffield, Sheffield, S1 4DE UK; 2 School of Engineering, University of Glasgow, Glasgow, G12 8LT UK; 3 EPSRC National Centre for III-V Technologies, University of Sheffield, Broad Lane, Sheffield, S3 7HQ UK; 4 Department of Electrical and Electronic Engineering, Graduate School of Engineering, Kobe University, 1-1 Rokkodai, Nada, Kobe, 657-8501 Japan

**Keywords:** Nonlinear optics, Infrared spectroscopy, Terahertz optics

## Abstract

A broadly tunable THz source is realized via difference frequency generation, in which an enhancement to χ^(3)^ that is obtained via resonant excitation of III–V semiconductor quantum well excitons is utilized. The symmetry of the quantum wells (QWs) is broken by utilizing the built-in electric-field across a p–i–n junction to produce effective χ^(2)^ processes, which are derived from the high χ^(3)^. This χ^(2)^ media exhibits an onset of nonlinear processes at ~4 W cm^−2^, thereby enabling area (and, hence, power) scaling of the THz emitter. Phase matching is realized laterally through normal incidence excitation. Using two collimated 130 mW continuous wave (CW) semiconductor lasers with ~1-mm beam diameters, we realize monochromatic THz emission that is tunable from 0.75 to 3 THz and demonstrate the possibility that this may span 0.2–6 THz with linewidths of ~20 GHz and efficiencies of ~1 × 10^–5^, thereby realizing ~800 nW of THz power. Then, transmission spectroscopy of atmospheric features is demonstrated, thereby opening the way for compact, low-cost, swept-wavelength THz spectroscopy.

THz spectroscopy is a powerful analytical technique for chemical and gas sensing, such as the detection of explosives and related compounds^1,2^, even in sealed containers^3^. The realization of suitable sources has recently been the subject of intense research^4^, yet remains a challenge. Fourier-transform infrared (FTIR) spectroscopy^5^ and THz time-domain spectroscopy (TDS)^6^ are commonly employed techniques^7^. Swept-wavelength spectroscopy has recently been identified as having significant signal-to-noise advantages over FTIR, thereby offering a route to high-throughput spectroscopic screening and sensing^8^. For application in a swept-wavelength spectrometer, broad tunability, narrow spectral linewidth, and sufficiently high power are required of the source, in addition to the requirements of efficiency, simplicity and robustness.

Here, we describe the generation of CW monochromatic, tunable THz waves via second-order nonlinear optical effects (difference frequency generation (DFG)) in GaAs/AlAs QWs and the realization of a THz transmission spectrometer. Figure 1 schematically illustrates our approach. χ^(3)^ is enhanced via resonant excitation of III–V semiconductor quantum well excitons^9^. Symmetry breaking of the quantum well is realized via the built-in electric field across a p–i–n junction to produce effective χ^(2)^ processes^10^. The comparatively low-power density threshold for the onset of nonlinear effects and the realization of phase matching laterally through normal incidence excitation enables area scaling of the THz emitter. The use of overlapping heavy-hole and light-hole exciton resonances enables emission over a broad spectral range of (0.2–6 THz). Finally, we demonstrate absorption spectroscopy of an atmospheric water absorption peak at 750 GHz.

**Fig. 1:**
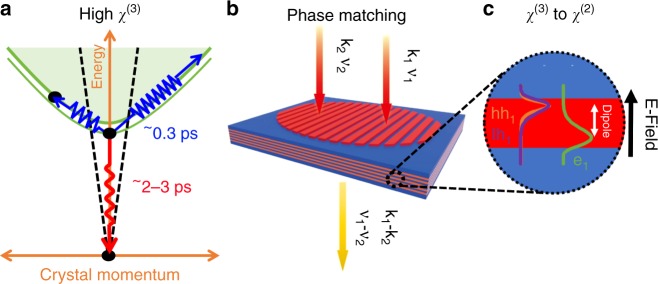
Schematic diagram for our broad spectral bandwidth THz emitter. High χ^(3)^ is obtained (**a**) via resonant excitation of excitons in a semiconductor quantum well. Phase matching is realized in-plane via excitation in normal incidence (**b**), which yields high-χ^(2)^ processes via the application of an internal electric field (**c**)

QW excitons are a 2D coherent elementary excitation, which give rise to a macroscopic transition dipole moment, that have been predicted to exhibit high χ^(3)^ optical nonlinearities due to the combination of this macroscopic transition dipole moment and the rapid radiative decay of the excitons^9^. Figure 1a illustrates the dispersion of excitons and e–h pairs. Upon resonant excitation^11^, the exciton may recombine radiatively (~2–3 ps)^12^ or, more likely, be thermally ionized via phonon scattering (~0.3 ps at room temperature)^13^. The rapid radiative decay and thermal ionization of the excitons pushes the exciton away from behaving as an ideal Boson, thereby enhancing χ^(3)^. Further macroscopic enhancement of χ^(3)^ by a factor of (*L*/*a*_B_)^2^ for near-resonant excitation (*L* denotes the coherently excited QW area and *a*_B_ the exciton radius) is possible if the spatial coherency of the pump light and exciton is maintained^9^. As this effect utilizes the imaginary part of χ^(3)^, which corresponds to absorption-saturation/state-filling effects, Coulomb enhancement of χ^(3)^ effects may also occur due to screening, band-gap renormalization, and Coulomb interactions^14^.

Symmetry breaking results in the realization of effective χ^(2)^ processes from high χ^(3)^ materials. This can be achieved via the application of an electric field, such as that found at a semiconductor surface^10^ or within a p–n junction^15^. The third-order nonlinear susceptibility, namely, χ^(3)^, is converted to χ^(2)^ as the dipole moments orient themselves in the direction of the applied field, thereby enabling many important second-order nonlinear processes^10^. Electric-field-induced second harmonic generation (SHG) was initially used for the extraction of semiconductor material parameters from semiconductors^16^ and field measurements of silicon metal–oxide–semiconductor (MOS) transistor interfaces^17^. Here, we utilize this conversion process for the conversion of χ^(3)^ to χ^(2)^ in a resonantly excited exciton system.

Figure 1b shows a schematic diagram of the multi-QW structure for converting χ^(3)^ to χ^(2)^ processes, which is designed such that e_1_hh_1_ and e_1_lh_1_ resonances are accessible by commercially available semiconductor lasers that operate at ~850 nm. Additionally, overlapping excitonic transitions enable a contiguously broad tuning range for difference frequency generation. The built-in E-field that is due to the unbiased p–n junction (26 kV cm^−1^) is sufficient for providing electron and hole wave-function separation of 7 nm, which is comparable to the QW width; see S1 for details of the device band-structure and the characterization of the excitonic absorption. For this proof-of-concept work, a detailed study of the optimal QW E-field and QW number for high efficiency/broad tuning range has not yet been conducted.

Following epitaxial growth of the structure and fabrication into a suitably mounted optical component, it was inserted into a measurement system as detailed in S2. Normal incidence of the two semiconductor lasers results in the generation of a coherent exciton grating within the χ^(2)^ material. Therefore, the requirements for phase matching of the two pump lasers are satisfied laterally, in contrast to the phase-matching requirements in waveguided nonlinear optical devices^18^.

Figure 2a presents the absorption spectrum of the sample and a schematic diagram of the laser excitation scheme in our experiments. The absorption spectrum (solid red line), which was obtained using a low-power, spectrally resolved white-light source (2.5 × 10^−4^ W cm^−2^), shows an electron-heavy hole exciton (e_1_hh_1_ at 1.453 eV) and electron-light hole exciton (e_1_lh_1_ at 1.461 eV) transition that is superimposed upon the step-like 2D density of states^19^. The e_1_hh_1_ (e_1_lh_1_) exciton binding energy is predicted to be 10.5 meV (13.5 meV). Direct excitation of the e_1_hh_1_ (e_1_lh_1_) density of states is expected at 1.464 meV (1.475 meV)^20^. The transmission data (blue points) at the maximum output power (68 W cm^−2^) of the excitation lasers are essentially identical; hence, heating of the sample is limited and the excitation power density has not bleached the excitonic absorption. A density of 3.8 × 10^9^ cm^−2^ excitons per layer is deduced by assuming a 1 ns carrier lifetime. An exciton density of 7 × 10^10^ cm^−2^ excitons per layer has been demonstrated to support excitonic absorption in similar samples^13^. The coincidence of the high- and low-power absorption spectra also demonstrates that there is no significant reduction in the built-in field due to photovoltages that are caused by absorption and carrier accumulation at the doped regions.

**Fig. 2:**
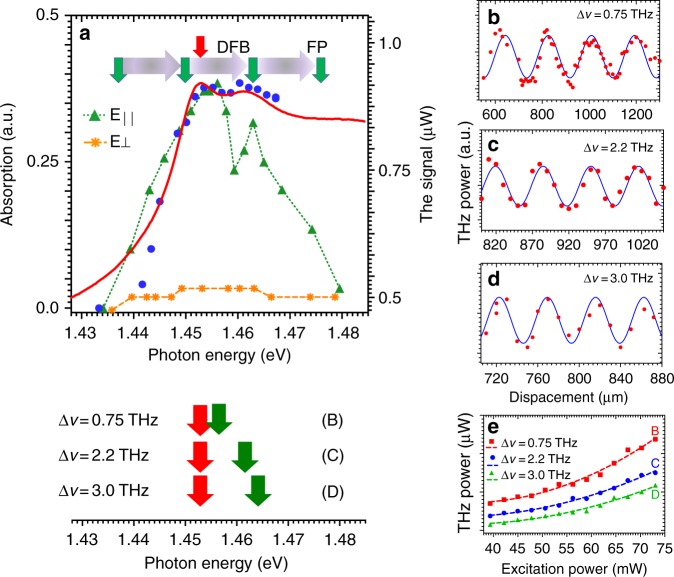
Absorption spectrum that was obtained using low incident powers (red) and high incident powers (blue dots) for the multi-quantum well structure. **a** The detected THz power using co-linear (green) and crossed polarized light (yellow) is plotted as a function of the energy of the tuneable laser. The calculated energies of the 1S e_1_hh_1_ and e_1_lh_1_ 1S exciton and interband absorption are also plotted. The power as a function of the etalon separation is plotted for difference frequencies of **b** 0.75 THz, **c** 2.2 THz, and **d** 3.0 THz. The power dependence of the measured signal at these frequencies is plotted in **e**

Figure 2a plots the measured THz power that was obtained in our system with both pump lasers operating at 130 mW as a function of the energy of the frequency tunable Fabry-Pérot (FP) laser with the other distributed feedback (DFB) laser resonant with the e_1_hh_1_ exciton. A Gentec-EO THZ-I-BNC detector was used to detect the THz radiation. It has a responsively of 10^5^ V/W at 0.63 μm.

The THz power is measured for the lasers in collinear and crossed polarizations. A clear signal is observed for collinearly polarized pump sources that follows the envelope of the exciton resonances. The polarization of the emitted THz has not been measured. Direct excitation of the 2D density of states is not expected in the region where a THz signal is generated. The difference with the case of crossed polarization is marked, with no strong exciton-state-related peak being observed. For collinearly polarized light, a (coherent) population grating creates our second-order nonlinear media (Fig. 1b) with decay times of (2 ps) 1 ns. For crossed polarization of the incident light, a spin grating is expected to be created, with a decay on an ~50 ps timescale^21^. The fast decay of this spin grating compared to the population grating may explain the dependence of the THz signal on the polarization of the pump lasers. Further details on the excitonic features and room-temperature excitonic absorption are provided in S3.

The lower section of Fig. 2a shows a schematic diagram of the excitation schemes for the interferometric measurement of wavelength for the data that are shown in Fig. 2b–d and described in detail in S4. The experimental data in Fig. 2b–d are fit with solutions to the Airy distribution for the FP resonator that was used. Monochromatic emission is confirmed from 0.75 to 3.0 THz; however, the low finesse of the resonator prevents confirmation of the linewidth of the emission. The linewidth of single-mode semiconductor lasers can be expected to be ~1 MHz, thereby leading to similar expected linewidths of the THz emission from DFG.

Figure 2e plots the THz power as a function of the excitation power (pump power × absorption) at 0.75, 2.0, and 3.0 THz. A clear quadratic relationship is observed, thereby confirming that second-order nonlinear effects are observed. For the peak in the THz power at ~0.75 THz, a conversion efficiency of 1.2 × 10^−5^ is measured. This compares highly favorably with other difference frequency generation (DFG)-based THz generation results^18^ and has the advantage of wide tunability. Measurements of the THz power as a function of laser detuning are presented in S5. These show that emission frequencies 0.2 to 6.5 THz may be possible based on the confirmation of emission frequencies from 0.75 to 3 THz.

According to simple calculations that are based on the observed conversion efficiency^22^, the second-order susceptibility is estimated to be ~2 nm/V. For the applied electric field of 26 kV cm^−1^ and χ^(2)^ = 3χ^(3)^*E*_dc_^15^, the magnitude of the third-order susceptibility of the resonantly excited excitonic resonance is ~2.6 × 10^−16^ m^2^ V^−2^. This is ~500 times that expected for bulk GaAs at room temperature in the region of the band-edge, ignoring resonant excitonic effects^23^. Hanamura^9^ predicted a low-temperature (i.e., zero-dephasing-rate) resonant enhancement of (*L*/*a*_B_)^2^, where *L* is the coherent area of excitation (assumed to be the wavelength of the excitation) and *a*_B_ the exciton Bohr radius, which yields an enhancement of ~9 × 10^4^. The observed enhancement by ~500 times at room temperature is highly attractive and we hope this will encourage further experimental and theoretical studies of this system, e.g., on the effects of rapid dephasing on the χ^(3)^ enhancement, the spatial extent of the exciton coherence, and the screening of excitonic effects by the electron-hole plasma. The experimental data exhibit rich behavior. For example, the comparative strength of the THz generation with light-hole excitation is large despite 1/3 excitation strength due to selection rules^24^ and ~1/2 oscillator strength^20^ compared to heavy-hole exciton excitation. The THz generation is high for small difference frequencies, which may demonstrate the presence of more than one THz generation process.

The conversion efficiency and output power are not yet maximized in these proof-of-concept measurements. To optimize the conversion efficiency, an optimal E-field should be employed that balances the ability to readily convert χ^(3)^ to χ^(2)^ processes and the reduction of absorption into the exciton state due to the Stark effect. Furthermore, the excitation density is not yet optimized with regard to conversion efficiency, with saturation and bleaching of the exciton population yet to be demonstrated. It is our expectation that higher conversion efficiencies than the realized value of 1.2 × 10^−5^ are possible. Once conversion efficiency has been maximized, area scaling and an increase in the number of QWs should be employed to enhance the output power. Considering the availability of semiconductor-based tunable lasers and fiber amplifiers, compact tunable THz sources with ~mW power levels are expected in future, which will open up exciting opportunities in swept-source THz spectroscopy.

Figure 3 plots the attenuation coefficient of the THz signal through air between 650 and 850 GHz, which spans a water absorption line at 750 GHz. The details of the experimental apparatus are outlined in S6. The experimental data are plotted, in addition to the expected atmospheric absorption values that are valid for this frequency range, which were computed by summing individual absorption lines^25^. Also plotted is the convolution of this expected absorption spectrum with a Gaussian function that is equal to the line-shape broadening that is expected due to the non-ideality of one of the pump lasers. Excellent agreement of the peak position between the measured data and the expected values^25^ is observed, thereby demonstrating the utility of this tunable THz source in performing absorption spectroscopy. DFB lasers should provide ~ < MHz linewidths, while external cavity lasers can provide < kHz linewidths. Future optimized systems should have resolutions that are similar to these values (notwithstanding a future trade-off between linewidth and sweep speed^26^). This compares very favorably with the ~few-GHz pressure-broadened lines for atmospheric pressure gases and biological samples. Such a source may also be realized in a compact module that is suitable for deployment in a wide range of applications.

**Fig. 3:**
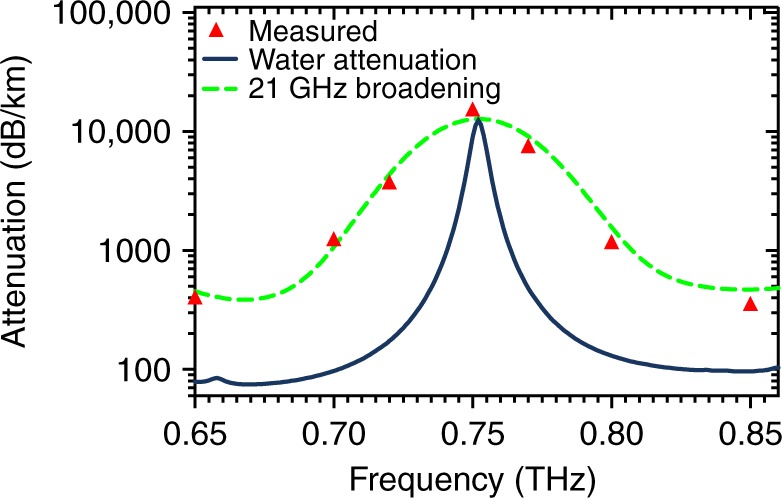
Measured atmospheric absorption coefficient values (red triangles) with the simulated water absorption spectrum (blue) and a modeled spectrum (green) that is broadened by the expected spectral linewidth of the Thz source

In summary, we have described the generation of CW monochromatic, tunable THz waves via second-order nonlinear optical effects in GaAs/AlAs QWs and the realization of a THz transmission spectrometer. We utilized an enhancement of χ^(3)^ via resonant excitation of III–V semiconductor quantum well excitons and broke the crystalline symmetry of the quantum wells by utilizing the built-in electric field across a p–i–n junction to produce effective χ^(2)^ processes. Phase matching laterally, through normal incidence excitation, enabled simple alignment of the system and area scaling of the THz emitter to yield ~µW power levels. The use of both heavy-hole and light-hole exciton resonances enables these power levels to be achieved over a broad spectral range that covers ~200 GHz to ~6 THz. We have demonstrated absorption spectroscopy of an atmospheric water absorption peak at 750 GHz and have discussed the resolution and power levels that will be attainable in future spectroscopy systems.

## Methods

### Sample preparation

The sample was grown via solid-source molecular beam epitaxy (MBE) on an n + GaAs (100) substrate using standard growth methods. The epitaxial sequence of the structure consisted of 200 nm n-doped GaAs (1 × 10^18^ cm^−3^), followed by 1500 nm, n-doped Al_0.4_Ga_0.6_As (5 × 10^17^ cm^−3^). This is followed by the deposition of the MQW, which was made up of 30 repeats of 11.9 nm undoped GaAs quantum wells that were separated by 7.1 nm undoped AlAs barriers. Subsequently, 1500 nm p-doped Al_0.4_Ga_0.6_ As (5 × 10^17^ cm^−3^) was grown. The epitaxy was completed by growing 200 nm p-doped GaAs (1 × 10^19^ cm^−3^). Therefore, the multi-QW structure consists of a 30× period AlAs/GaAs MQW that is completed by n- and p-doped Al_0.4_Ga_0.6_As layers to induce a built-in E-field (*E* = 26 kV cm^−1^). A single excitonic transition has a linewidth of only ~10 meV at RT, while our structure, which as two overlapping excitonic resonances, is designed to have excitonic transitions that span ~20 meV.

Following epitaxy, the p + GaAs cap and GaAs substrate were removed. Initially, a selective GaAs etch was used to remove the p + cap. The sample was capillary bonded to a diamond heat-spreader and a combination of polishing and wet etching was used to remove the GaAs substrate. Then, the heat-spreader was bonded to a copper holder for insertion into the optical system.

## Supplementary information

Supplementary Information
