# Supplementary material for: Broadband THz absorption spectrometer based on excitonic nonlinear optical effects
Source: Light Sci Appl. 2019 Mar 13;8:29. doi: 10.1038/s41377-019-0137-y (PMC6414654; doi:10.1038/s41377-019-0137-y)
Supplement: Supplementary file 1 — Supplementary Information [file 41377_2019_137_MOESM1_ESM.docx]

**“Broadband THz Ansorption Spectrometer Based on Excitonic Nonlinear Optical Effects”**

**Supplementary Information**

Avan Majeed^1^, Pavlo Ivanov^2^, Benjamin Stevens^1^, Edmund Clarke^3^, Iain Butler^2^,

David Childs^2^, Osamu Kojima^4^, Richard Hogg^2^

*^1^ Department of Electronic and Electrical Engineering, University of Sheffield, Sheffield, S1 4DE, U.K.*

*^2^ School of Engineering, University of Glasgow, Glasgow, G12 8LT, U.K.*

*^3^ EPSRC National Centre for III-V Technologies, University of Sheffield, Broad Lane, Sheffield, S3 7HQ, U.K.*

*^4^ Department of Electrical and Electronic Engineering, Graduate School of Engineering, Kobe University, 1-1 Rokkodai, Nada, Kobe 657-8501, Japan*

**S1. Sample Band-Structure & Exciton Characterisation**

Supplementary Fig. 1 plots a schematic of the band-structure of the sample following substrate and p+ cap removal, calculated using a self-consistent 8 band k.p model. For our sample, an electric field of 26 kVcm*^-1^* is expected within the quantum wells. Solutions to Schrödinger’s equation allow the wavefunctions and transition energies of the quantum well excitonic transitions to be determined.

**Supplementary Figure 1.** Schematic of the band-structure of our MQW structure.

The as-grown epitaxial material was processed into 400 μm diameter annular diodes with a central 200 μm window for optical access. Supplementary Fig. 2(a) plots the photocurrent spectra obtained at a range of reverse biases in order to explore the Stark-effect in our device. The spectra are constituted of an e_1_hh_1_ exciton at 1.453 eV, and e_1_lh_1_ exciton at 1.461 eV, superimposed upon the step-like 2D density of states. The e_1_hh_1_ (e_1_lh_1_) exciton binding energies are predicted to be 10.5 mev (13.5 meV). Direct excitation of the e_1_hh_1_ (e_1_lh_1_) density of states is expected at 1.464 meV (1.475meV). Upon the application of a reverse bias, both transitions exhibit a redshift. At the highest bias, a feature at 1.47 eV emerges, which is attributed to the e_1_hh_2_ excitonic transition, which whilst forbidden at zero bias, has finite electron-hole overlap at higher bia.

**
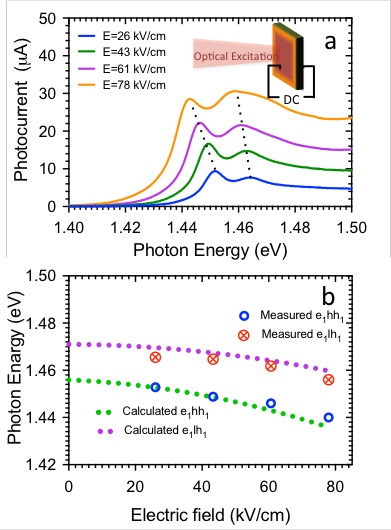
**

**Supplementary Figure 2.** (a) Photocurrent spectra for an optical access mesa-diode fabricated from the as-grown epitaxial structure. (b) Measured excitonic transition peak energies, and simulated transition energies.

Figure 2(b) plots the measured e_1_hh_1_ and e_1_lh_1_ excitonic transition energies from the photocurrent spectra as a function of electric field within the QW. Also plotted are the modelled excitonic transition energies.

**S2. THz generation and detection system**

The sample, bonded to a heat-spreader and attached to a copper holder was inserted into the optical system. See Supplementary Figure 3. Two lasers, one (DFB) tuned to the e1-hh1 absorption peak and the other (F-P) tuned across the absorption band-edge of the MQWs. The two lasers were collimated and directed co-linearly normal to the sample surface with a beam spot size of ~0.7 mm. An ND filter wheel allowed simultaneous control of both excitation lasers, and a number of pellicle beam splitters allowed the illumination and imaging of the sample, in addition to sampling the two lasers emission spectra. A Si lens was mounted on the copper sample holder and a long-pass filter (TPX) ensured that only emission >20 um was incident upon the pyro-electric detector. One of the lasers was modulated (2Hz) to allow standard lock-in techniques to be employed to recover the generated THz signal intensity.

**
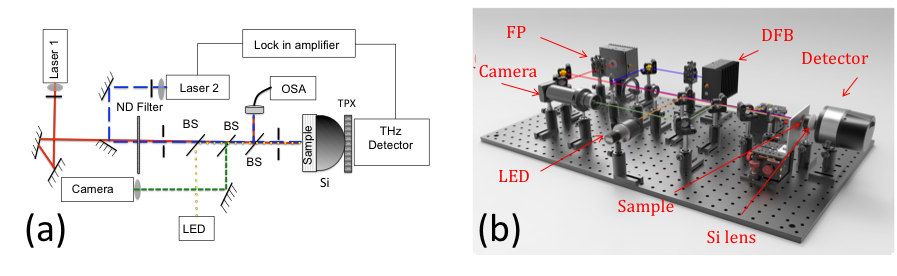
**

**Supplementary Figure 3.** Schematic (a) and rendered image (b) of experimental apparatus for THz generation and detection.

**S3. Room Temperature Excitonic Absorption Processes & Spectrum**

The room temperature absorption spectrum for our sample is shown in supplementary figure 4. A fit to the absorption spectrum is made using calculated e_1_hh_1_ and e_1_lh_1_ excitonic, and 2D e-h absorption edge transitions. The excitonic and band-edge transitions are broadened after Chemla, *et al.,* [11].

We note that the coincidence of this absorption spectrum with the photocurrent shown in supplementary figure 2(a) indicates that substrate and p+ cap removal have not modified the electric field within the MQW, as removal of the electric field would result in a ~3 meV blue-shift in the absorption peak position (through both simulation and quadratic fit to experimental data).

**Supplementary Figure 4**. Absorption spectrum of the MQW sample, with fit to the spectrum (see text).

At room temperature, the exciton is in a special situation in terms of energies within the system:

**Phonon (*ħ*Ω_LO_)> Thermal (kT_B_)> Binding (B)> Exciton Linewidth (Γ)**

As *ħ*Ω_LO_)> B, the scattering of an exciton with an LO Phonon is expected to result in the ionisation of the exciton into a free electron and hole. As kT_B_**>** B the formation of excitons from these electrons and holes is highly unlikely. This leads to the short exciton lifetime and high χ^(3)^ (e.g. Reference 9 in main paper) and subsequent χ^(2)^ ((e.g. Reference 15 in main paper) that we exploit here.

**S4. Spectral Measurements**.

A Fabry-Perot interferometer (FPI) was formed within the structure described above by inserting two semi-insulating silicon wafers (one fixed, one translated, 4” diameter, single-side polished) between the THz emitter and the detector. See supplementary figure 5.


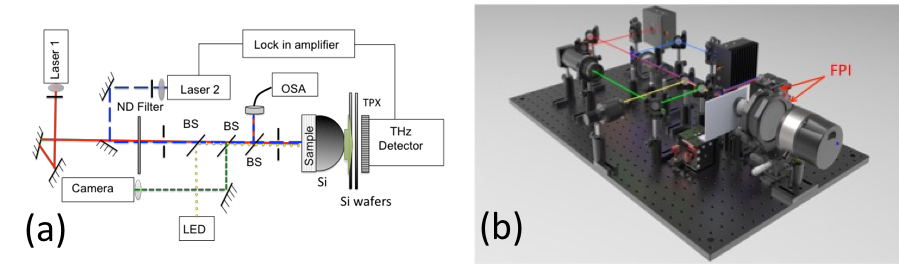


**Supplementary Figure 5**. Schematic (a) and rendered image (b) of experimental apparatus, showing position of Fabry-Perot interferometer.

The transmittance function of the Fabry-Perot interferometer is given by;

$$T=\frac{1}{1+F \sin^{2}(\frac{\delta}{2})}$$

Where T is the transmittance, F is the resonator finesse, and δ the phase difference between each successive transmitted pair. δ is given by;

$$\delta=\frac{2\pi}{\lambda}2n\mathcal{l}\cos\left( \phi\right)$$

Where c is the speed of light in vacuum, φ is the incident angle of the light into the interferometer, l is the separation of the interferometer mirrors, n is the refractive index of the material between the two reflecting surfaces.

The coefficient of finesse of the interferometer is calculated from;

$$F=\frac{4R}{{(1-R)}^{2}}$$

Where R is the reflectance;

$$R=\left( \frac{n_{1}-n_{2}}{n_{1}+n_{2}} \right)^{2}$$

Where n1 is refractive index of silicon, and n2 is the refractive index of air.

The refractive index of the Si ≈ 3.41 [1], and the reflectance R is calculated to be 29% under normal incidence.

The THz output wavelengths were measured by scanning the Si-based interferometer. Fig. 2 B, C, D show the signals detected for the three different excitation cases as a function n of translation stage position for one of the etalon mirrors. Fits were made using the experimentally measured resonator mirror separation.

**S5. Range of Possible Emission Frequencies**

Supplementary figure 6 plots the measured THz power as a function of laser detuning, both positive and negative with respect to the DFB laser (1.453eV). Data is highlighted that shows confirmed emission frequencies through the aforementioned spectral measurements.


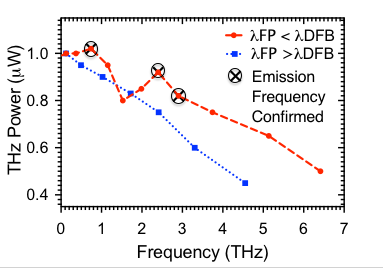


**Supplementary Figure 6**. THz power as a function of predicted THz emission frequency obtained by detuning the FP laser w.r.t. the DFB laser. Experimentally confirmed emission frequencies (Fig 2) are marked.

**S6. Absorption Spectrometer**.

In order to convert the apparatus described in S1 into an absorption spectrometer, the detector mount was modified. A parabolic mirror collimated the THz emitter output. Another parabolic mirror and the THz detector were mounted on a translation stage to enable the absorption path-length in air to be varied. For a given laser detuning (and hence different THz frequency) the path length was increased by 100mm and attenuation of the signal calculated from these two absorption lengths (50mm and 150mm). See Supplementary figure 7.


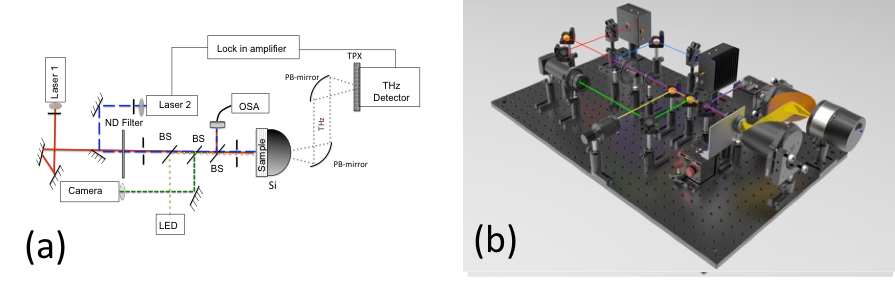


**Supplementary Figure 7.** Schematic (a) and rendered image (b) of experimental apparatus showing the creation of a variable absorption path for absorption spectroscopy.

The bandwidth of the emitted THz radiation is expected to be governed by the linewidth of the pump laser sources. Spectral measurements showed a measurement resolution limited linewidth of the DFB laser, but an unexpected broadening of the FP laser of ~21GHz [2]. The origin of this spectral broadening is not known. The fit to experimental data in Fig. 3 was made by convoluting the expected water transmission spectrum (blue) with a 21GHz Gaussian function (green). A very similar fit was obtained using a Lorentzian function, but a Gaussian function was used as the origin of the broadening is not known.

**References**

[1] Li, H.H.: Refractive-index of silicon and germanium and its wavelength and temperature derivatives. J. Phys. Chem. Ref. Data 9, 561–658 (1980).

[2] C.H. Henry “Theory of the Linewidth of Semiconductor Lasers”. IEEE Journal

of Quantum Electronics, 18(2), 259, (1982)
